# Dicer regulates *Xist *promoter methylation in ES cells indirectly through transcriptional control of Dnmt3a

**DOI:** 10.1186/1756-8935-1-2

**Published:** 2008-10-27

**Authors:** Tatyana B Nesterova, Bilyana C Popova, Bradley S Cobb, Sara Norton, Claire E Senner, Y Amy Tang, Thomas Spruce, Tristan A Rodriguez, Takashi Sado, Matthias Merkenschlager, Neil Brockdorff

**Affiliations:** 1 Developmental Epigenetics Group, MRC Clinical Sciences Centre, Faculty of Medicine ICSTM, Hammersmith Hospital, Du Cane Road, London, UK; 2 Lymphocyte Development Group, MRC Clinical Sciences Centre, Faculty of Medicine ICSTM, Hammersmith Hospital, Du Cane Road, London, UK; 3 Molecular Embryology Group, MRC Clinical Sciences Centre, Faculty of Medicine ICSTM, Hammersmith Hospital, Du Cane Road, London, UK; 4 Division of Human Genetics, National Institute of Genetics, Research Organization of Information and Systems, 1111 Yata, Mishima, 411-8540, Japan; 5 Department of Biochemistry, University of Oxford, South Parks Road, Oxford, UK

## Abstract

**Background:**

X chromosome inactivation is the mechanism used in mammals to achieve dosage compensation of X-linked genes in XX females relative to XY males. Chromosome silencing is triggered in *cis *by expression of the non-coding RNA *Xist*. As such, correct regulation of the *Xist *gene promoter is required to establish appropriate X chromosome activity both in males and females. Studies to date have demonstrated co-transcription of an antisense RNA *Tsix *and low-level sense transcription prior to onset of X inactivation. The balance of sense and antisense RNA is important in determining the probability that a given *Xist *allele will be expressed, termed the X inactivation choice, when X inactivation commences.

**Results:**

Here we investigate further the mechanism of *Xist *promoter regulation. We demonstrate that both sense and antisense transcription modulate *Xist *promoter DNA methylation in undifferentiated embryonic stem (ES) cells, suggesting a possible mechanistic basis for influencing X chromosome choice. Given the involvement of sense and antisense RNAs in promoter methylation, we investigate a possible role for the RNA interference (RNAi) pathway. We show that the *Xist *promoter is hypomethylated in ES cells deficient for the essential RNAi enzyme Dicer, but that this effect is probably a secondary consequence of reduced levels of *de novo *DNA methyltransferases in these cells. Consistent with this we find that Dicer-deficient XY and XX embryos show appropriate *Xist *expression patterns, indicating that Xist gene regulation has not been perturbed.

**Conclusion:**

We conclude that *Xist *promoter methylation prior to the onset of random X chromosome inactivation is influenced by relative levels of sense and antisense transcription but that this probably occurs independent of the RNAi pathway. We discuss the implications for this data in terms of understanding *Xist *gene regulation and X chromosome choice in random X chromosome inactivation.

## Background

X chromosome inactivation is the mechanism used in mammals to achieve dosage compensation of X-linked genes in XX females relative to XY males. Early in development all cells in female embryos inactivate most genes on one of the two X chromosomes. In embryonic lineages X inactivation is normally random, with an equal probability of either the maternal or paternal X undergoing X inactivation in any given cell. In certain extraembryonic lineages it is always the paternal X that is inactivated, referred to as imprinted X inactivation. Following the establishment of X inactivation in early embryogenesis the inactive state is stably maintained through all subsequent cell generations (reviewed in [1]).

X inactivation is triggered by the expression of the X inactive specific transcript (*Xist*), an unusual non-coding RNA that has the unique property of binding to and coating the chromosome from which it is transcribed. *Xist *RNA is thought to recruit silencing factors that modify the chromatin, bringing about a mitotically stable heterochromatic configuration that can be propagated through subsequent cell divisions (reviewed in [2]).

Establishing appropriate X inactivation patterns requires mechanisms that ensure correct regulation of *Xist *RNA in early development. Specifically *Xist *expression must occur from only one allele in XX cells and not at all in XY cells. Studies to date indicate that regulation of *Xist *expression in random X inactivation is complex. An antisense RNA, termed *Tsix*, is important for maintaining the *Xist *gene in a poised state prior to the onset of X inactivation through a mechanism that is thought to involve establishment of repressive chromatin marks and/or DNA methylation over the *Xist *promoter [3-6]. *Tsix*-mediated repression in turn regulates the probability of a given *Xist *allele being expressed in XX heterozygotes [7-11]. Concurrent with *Tsix *expression there is a low level of sense transcription from the *Xist *promoter [6,12]. Enhanced sense transcription initiated from ectopic sites upstream of *Xist *antagonises *Tsix *and renders that chromosome more likely to be inactivated in XX heterozygotes [12,13].

In undifferentitated embryonic stem (ES) cells, the absence of *Tsix *expression or of DNA methylation leads to only a modest increase in *Xist *expression [8,9,11,14]. Conversely, following the onset of cellular differentiation, both *Tsix*-deficient and DNA methylation-deficient ES cells upregulate *Xist *inappropriately, that is, from the single X chromosome in XY cells [7,14-17]. This suggests the existence of alternative, possibly redundant mechanisms for regulating *Xist *expression. Indeed studies on X chromosome reactivation in the inner cell mass [18,19], and also in developing primordial germ cells [20], suggest that an overlapping regulatory pathway, specific for pluripotent cells (including ES cells), either represses *Xist *transcription, or alternatively reduces levels of a critical positive regulator of *Xist *expression.

Interwoven into this complex regulatory circuitry there is an additional pathway that ensures only a single *Xist *gene is expressed in XX cells, and that the single *Xist *allele in XY cells remains repressed. The classical model for this process invokes the presence of a blocking factor present at limiting levels such that only a single *Xist *allele is blocked in each cell [21]. More recently evidence has emerged that *trans*- interactions between *Xist *alleles are important in the allelic control of *Xist *expression [22-24]. Also, in a recent study it has been proposed that the gene encoding a critical positive regulator is located close to *Xist*. Increased levels of this factor in the cells of early embryos with more than one X chromosome are proposed to attain a threshold level that allows expression of *Xist *[25].

The RNA interference (RNAi) pathway is found in organisms as diverse as fission yeast and mammals (reviewed in [26]). The pathway governs a range of mechanisms that regulate gene expression at the level of RNA translation/stability (post-transcriptional gene silencing) and at the level of transcription/chromatin structure (transcriptional gene silencing). Given the involvement of non-coding RNAs in X inactivation, a possible link with the RNAi pathway, either in initiation, propagation or maintenance of X inactivation, has been conjectured. A role for RNAi in the initiation of X inactivation in particular is suggested by the presence, prior to the onset of X inactivation, of overlapping sense and antisense RNAs at the *Xist *locus. We previously provided evidence that RNAi does not play a role in the maintenance of X inactivation using conditional deletion of the gene encoding Dicer, an RNase III enzyme that is essential for the RNAi pathway, in T-lymphocytes [27]. In this study we further explore the role of RNAi in the initiation and propagation of X inactivation. We show that both sense and antisense transcription across the *Xist *promoter influence levels of DNA methylation, consistent with a dsRNA-mediated mechanism. Using conditional *Dicer *knockout ES cells we demonstrate that *Dicer *deletion leads to hypomethylation of the *Xist *gene promoter. However, we also find that hypomethylation of the genome occurs more widely and is attributable to reduced levels of *de novo *methyltransferases, most notably Dnmt3a, in ES cells. Moreover, analysing Dicer-deficient embryos at E6.5 we demonstrate that the initiation of monoallelic *Xist *expression and spreading of *Xist *RNA occur normally. We conclude that the RNAi pathway does not play a critical role in the X inactivation process.

## Results

### Sense transcription across the *Xist *promoter influences *Xist *promoter methylation in undifferentiated ES cells

We and others have previously shown that sense as well as antisense transcription across the *Xist *locus prior to the onset of X inactivation plays a role in X chromosome inactivation choice [7-9,11-13]. The mechanism for this is unknown although there is evidence that *Tsix *has an influence on chromatin structure and DNA methylation of the *Xist *promoter.

**Figure 1:**
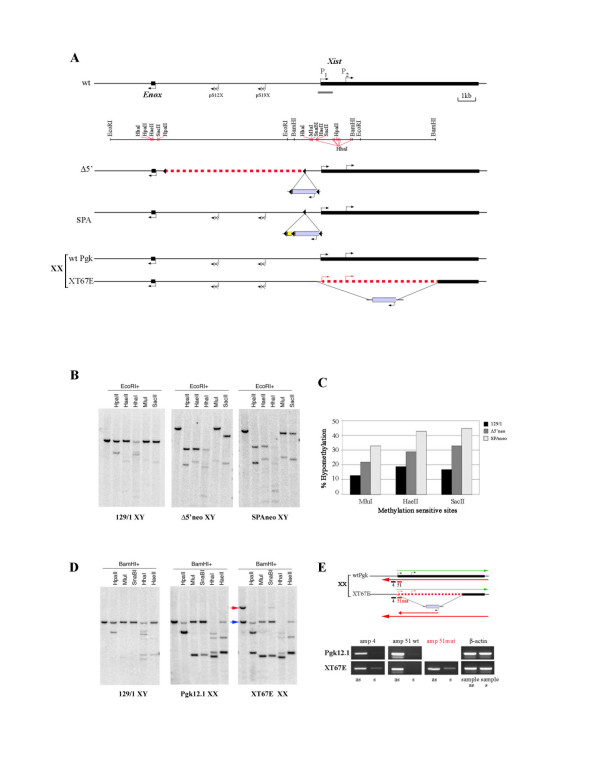
**Analysis of *Xist *promoter DNA methylation in *Xist *mutants**. (A) Schematic representation spanning *Xist *and the immediate upstream gene, *Enox*, including pS12x and pS19x. *Xist *and *Enox *TSS and the direction of transcription are indicated by arrows. The restriction methylation-sensitive enzymes used in the analysis are shown underneath the schematic. The grey bar shows the position of the probe used for Southern blot hybridisation. The three targeted *Xist *mutants Δ5', SPA [12] and XT67E1 [29] are shown. The dotted red line shows the deletions in Δ5' and XT67E1 mutants and the lilac box below the schematic represents an insertion of a floxed PGKneo cassette. The small yellow box shows the position of the SPA insertion. (B) MSRE analysis of the *Xist *promoter in wt (129/1) and two mutant (Δ5'+neo and SPA+neo) XY ES cell lines. The different sizes of parental EcoRI fragments in *Xist *mutants are due to deleted/inserted sequences. The increased intensity of digested fragments in mutant samples indicates partial hypomethylation. (C) Quantitation of the degree of hypomethylation of MluI, HaeII and SacII sites in wt and mutant cell lines. (D) MSRE analysis of the *Xist *promoter in wt XY (129/1), wt XX (Pgk12.1) and mutant (XT67E1) XX ES cell lines. The blue arrow indicates the methylated wt PGK fragment and the red arrow to the larger mutant XT67E1 fragment. Note the complete loss of DNA methylation in the *Xist *upstream region on the XT67E1 mutant allele. (E) Strand-specific RT-PCR analysis of the *Xist *5'region in wt Pgk12.1 and mutant XT67E1 XX ES cell lines. The position of the primers for amplicon 4 (amp 4), amplicon 51 (amp 51), amplicon 51mut (amp51mut) and the direction of sense (s, green) and antisense (as, red) transcripts are shown on the schematic above. Note the expression of ectopic sense transcript in XT67E1, attributable to the mutant allele.

Evidence to date indicates that antisense *Tsix *transcription has an influence on *Xist *promoter DNA methylation in differentiating ES cells [6], and in somatic cells [4,6,17], but not in undifferentiated ES cells that are representative of *Xist *promoter status prior to the onset of X inactivation [4,6,17]. To determine whether sense transcription can influence DNA methylation of the *Xist *promoter prior to X inactivation we analysed two XY ES cell lines carrying mutations in the *Xist *5' region, initially using conventional methylation sensitive restriction enzyme site (MSRE) analysis. The first mutation, Δ5', is a deletion of a 9 kb region 1.1 kb upstream of the *Xist *transcriptional start site (TSS). The second is an insertion of a transcriptional termination site SPA-MAZ_4 _in the region -1.1 kb relative to the TSS [12] (Figure 1A). Both of these mutations show enhanced sense transcription in undifferentiated XY ES cells correlating with preferential inactivation of the mutant X chromosome *in vivo *[12]. In accordance with previous findings [28], the *Xist *promoter was found to be highly methylated in undifferentiated wild-type (wt) XY ES cells (Figure 1A and 1B). Interestingly, both the Δ5'+neo and SPA+neo XY ES cell lines showed significant hypomethylation at all of the CpG sites analysed, that is, HpaII, HaeII, HhaI, MluI and SacII (Figure 1B). Quantification of the bands using ImageQuant software demonstrated methylation loss within a range of 20–45% for various CpG sites, with the SPA+neo mutant affected more severely (Figure 1C). This indicates that enhanced sense transcription across the *Xist *promoter can lead to CpG hypomethylation.

We then analysed another *Xist *mutation, XT67E1 [29], a deletion of the most part of *Xist *exon 1 and the minimal promoter region on the 129 allele in Pgk12.1 XX ES cells (Figure 1A). Whilst the deletion in XT67E1 cells removes a number of methylatable CpG sites in the *Xist *promoter, sites 36 bp upstream of the TSS are retained. Moreover, the deletion gives rise to a change in size of a BamHI fragment in the *Xist *5' region, and we were therefore able to discriminate between wt and mutant alleles (Figure 1A and 1D). MSRE analysis revealed complete hypomethylation on the mutant allele. The wt allele was mosaically methylated, similar to the parental XX ES cell line Pgk12.1 [29].

As the *Xist *TSS is deleted on the mutant allele in XT67E1 cells we anticipated that sense transcription would not be detectable but that antisense *Tsix *transcription would be unaltered. However, analysis by strand specific polymerase chain reaction (PCR) revealed unexpectedly that the mutant allele transcribes both sense and antisense RNAs and moreover that sense transcripts are abundant relative to the parental Pgk12.1 cell line (Figure 1E, amplicon 4). To verify this result we designed primers that were able to discriminate between mutant and wt alleles; the forward primer was the same for both alleles and was located 59–87 bp upstream of the *Xist *TSS. The reverse primers were located either in exon 1 (TN51, wt allele) or at the 3'end of the neomycin selective cassette (neoTN9 ≡ 51mut, mutant allele). Strand- and allele-specific reverse transcription (RT) PCR with these primers clearly demonstrates ectopic sense transcription on the mutant allele (Figure 1E). The PGK promoter that drives expression of the neomycin resistance gene cannot be the origin of ectopic transcripts as it is located about 1.7 kb downstream from the region analysed and in a reverse orientation relative to *Xist*. This suggests that sense transcription is activated from a minor upstream *Xist *TSS, in line with a result reported previously [13]. Importantly this result reinforces the conclusion from analysis of Δ5'+neo and SPA+neo mutant ES cells that enhanced sense transcription initiated upstream of *Xist *antagonises *Xist *promoter methylation.

### Hypomethylation of the Xist CpG island in undifferentiated ES cells correlates with X inactivation skewing

**Figure 2:**
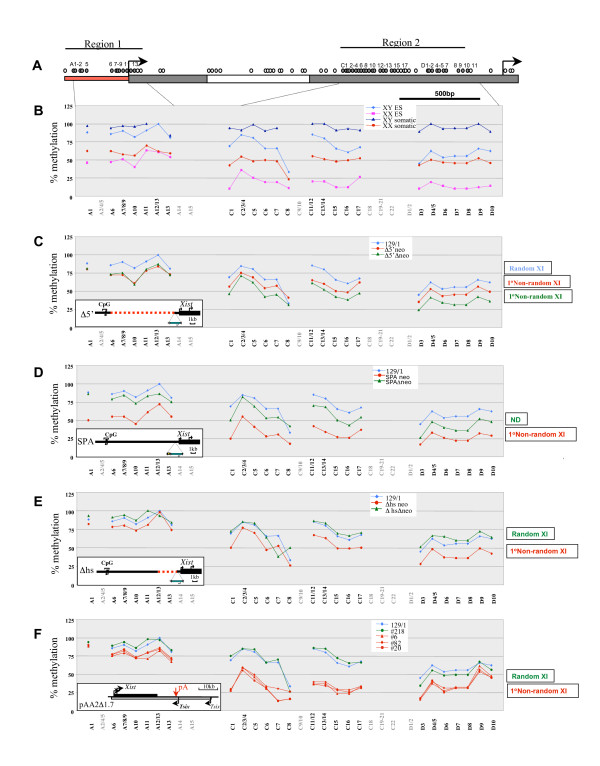
**SEQUENOM mass spectrometry analysis of *Xist *CpG island DNA methylation in *Xist *mutant XY embryonic stem cell lines**. (A) Schematic representation of the *Xist *promoter region and 5'end of exon 1 (CpG regions 1 and 2). The P1 and P2 start sites and the direction of transcription are indicated by arrows. The grey shaded box shows the position of the 5'repeats. Individual CpG sites are represented by small circles above the schematic; grey circles indicate the sites that were analysed. Polymerase chain reaction fragments A, C and D incorporate sites A1–15, C1–22 and D1–10 (see Methods). The graphs show the percentage of methylation of specific *Xist *CpG sites in wild-type (wt) XY and XX embryonic stem (ES) and somatic cells (B) and in Δ5' (C), SPA (D) and Δhs (E) *Xist *mutants [12,13] and in pAA2Δ1.7 and pSS1Δ2.7 (F) *Tsix *mutants [11]. The wt 129/1 XY ES cell line is included as a reference control on each graph. The insert shows the type and position of the mutation. X inactivation skewing phenotypes for each mutation are indicated alongside. The dots are joined by lines where consecutive sites were analysed. CpG sites numbered in grey below the graphs indicate that the data points are not available due to low or high fragment mass or due to duplication or overlay of two or more fragments. The average data for two or three CpG sites (for example, A7/8/9) is shown in cases when the sites reside close to each other and could not be resolved as separate fragments. Note the direct correlation between hypomethylation of the *Xist *promoter region in mutant ES cells and primary (1°) non-random X inactivation *in vivo*.

To investigate *Xist *promoter methylation in more detail we used SEQUENOM matrix-assisted laser desorption/ionisation time of flight (MALDI-TOF) mass spectrometry analysis of bisulphite-modified DNA [30]. This approach allowed us to analyse methylatable CpGs more widely and also to obtain accurate quantitative measurement of CpG methylation levels. To validate the method we first analysed the methylation patterns in control XX and XY somatic and ES cell lines (Figure 2A and 2B). As expected, CpG methylation was close to 100% in XY male somatic cells and about 50% in XX female somatic cells, representing the average of a fully methylated inactive *Xist *locus and a fully unmethylated active locus on the inactive X chromosome [28]. In XY ES cells methylation was close to 100% in *Xist *region 1, although somewhat lower in region 2. *Xist *was significantly hypomethylated in XX ES cells, consistent with our previous observations [31].

Having validated the SEQUENOM method we went on to analyse *Xist *promoter methylation for the mutant XY ES cell lines described above, and additionally in Δhs XY mutant ES cell lines where ectopic transcription-dependent skewing of X inactivation has also been reported to occur [13]. We found that deletion of 9 kb of the *Xist *upstream region (Δ5') leads to around 20% loss of methylation in both region 1 and region 2. This was also the case for mutants carrying the PGKneo cassette, as well as for Δneo ES cells (Figure 2C). More severe loss of methylation was observed for SPA+neo mutant (Figure 2D), consistent with the MSRE analysis above (Figure 1B and 1C). Moderate hypomethylation was observed for the Δhs+neo, but not for the Δhs Δneo mutant (Figure 2E). Strikingly, all of the mutants that show hypomethylation of the *Xist *CpG island also show preferential X inactivation of the mutant allele in female heterozygotes [12,13]. Conversely, the Δhs Δneo mutation that does not affect the randomness of X inactivation shows no hypomethylation. Collectively, these results demonstrate a direct correlation between hypomethylation of the *Xist *promoter and an increased probability of that chromosome being chosen as the inactive X in heterozygous females.

It was previously reported that abolition of *Tsix *transcription does not cause *Xist *promoter hypomethylation in undifferentiated ES cells, although analysis was restricted to two MSRE sites in region I [6]. To further address this issue we used the SEQUENOM assay to assess *Xist *promoter methylation in two different *Tsix *mutant ES cell lines, pSS1Δ2.7 and pAA2Δ1.7 [11]. In the first mutant, pSS1Δ2.7, *Tsix *exon 1 was deleted, but this had no effect on *Tsix *transcription or function. In the second mutant, pAA2Δ1.7, *Tsix *transcription through the *Xist *locus was abolished, causing primary non-random inactivation of the targeted allele in female embryos. We analysed three independent pAA2Δ1.7 XY ES cell clones, and in all cases we observed clear hypomethylation of the *Xist *promoter (Figure 2F). Hypomethylation in CpG region I was moderate compared with CpG region 2, possibly accounting for the fact that Sun et al. [6] did not observe this result. No methylation differences were observed for the pSS1Δ2.7 mutant, consistent with normal *Tsix *transcription and random X inactivation in female heterozygotes. This result suggests that prior to the onset of X inactivation, *Tsix *transcription, possibly together with physiological levels of sense *Xist *transcription, determines *Xist *promoter CpG methylation levels that, in turn, have an impact on the probability of that X chromosome being selected as the inactive X during the onset of random X inactivation. Increasing sense transcription, or possibly utilisation of a heterologous upstream promoter, antagonises promoter CpG methylation such that there is an increased probability of that X chromosome being selected as the inactive X in a heterozygous female.

### The role of the RNAi pathway in initiation of X inactivation: production of conditional Dicer knockout ES cells

What is the mechanism by which sense and antisense transcription impact on CpG methylation and repress the *Xist *promoter? One possibility is that sense and antisense RNAs trigger an RNAi response, similar for example to RNA-dependent DNA methylation in higher plants [26]. Alternatively, an RNAi-independent mechanism involving the antisense RNA *Tsix*, or both sense and antisense RNAs, may operate. The fact that increased sense transcription reduces *Tsix*-dependent *Xist *promoter methylation could be interpreted to indicate that silencing does not depend on dsRNA production. However, this does not rule out that the low levels of endogenous sense transcription from the *Xist *promoter co-operates with antisense *Tsix *RNA in RNAi-mediated silencing. To investigate this further we set out to analyse ES cells deficient for the RNase III enzyme Dicer, which is essential for the RNAi pathway in mammalian cells.

**Figure 3:**
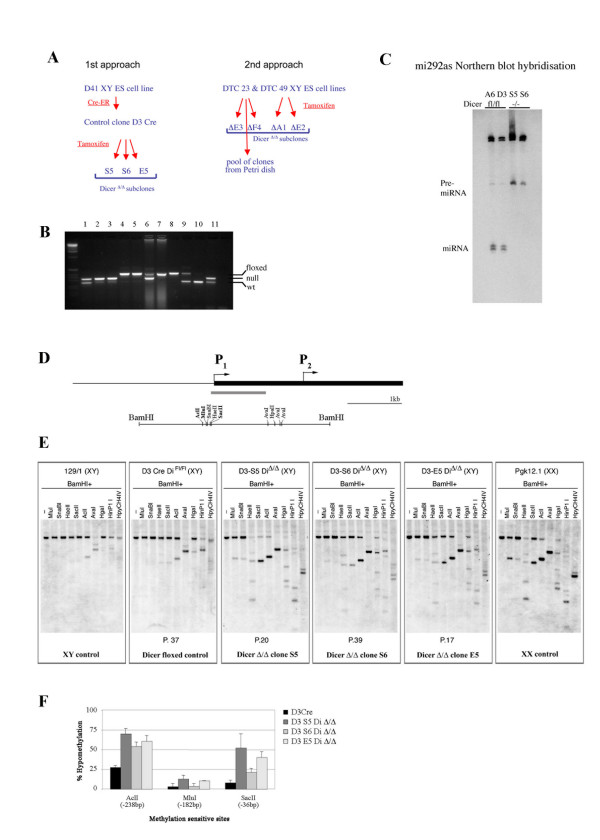
**Derivation and analysis of Dicer-deficient XY embryonic stem cell lines**. (A) Two approaches used to create Dicer-deficient embryonic stem (ES) cell lines (see Methods for details). (B) PCR genotyping assay to discriminate between *Dicer *wild-type (wt), floxed and deficient cell lines. 1–3 and 11, *Dicer *null clones; 4–5 and 7–8, *Dicer*^lox/lox ^parental cell lines; 6, a mixed clone with deleted and floxed alleles; 9, wt/Δ heterozygous mouse; 10, wt control. The wt band in Dicer-deficient clones is due to contamination of the ES sample with feeder cells. (C) Northern blot hybridisation of RNA from floxed cell lines (A6 and D3) and *Dicer *null clones (S5 and S6) with an mi292as probe to assay for Dicer function. Loss of miRNA and gain of pre-miRNA in S5 and S6 clones but not in floxed clones A6 and D3 indicate that Dicer function is abolished in mutant clones. (D) Schematic of the *Xist *5'region is shown alongside a restriction map. The grey bar indicates the position of the probe used for Southern blot hybridisation. (E) MSRE analysis of *Xist *promoter in control and mutant ES cell lines. DNA methylation level in *Dicer*^Δ/Δ ^clones is more similar to the hypomethylated XX cell line rather than the methylated XY control or parental XY floxed cell line. (F) Quantification of the degree of hypomethylation of *Acl*I, *Mlu*I, and *Sac*II sites in floxed and Dicer-deficient ES cell lines. The position of the sites relative to the *Xist *start site is shown in brackets.

We set out to derive an ES cell line in which the gene encoding Dicer can be deleted conditionally using CRE/loxP, allowing us to discriminate between primary effects attributable to *Dicer *deletion and secondary effects resulting from derivation and long-term culture of ES cells deficient for this essential factor. We established the D41 XY ES cell line, in which the RNase III domain was flanked with loxP sites on both *Dicer *alleles [27]. Initially, Dicer-deficient ES cell lines were derived from the D41clone by transfection with a pCAG-Mer-Cre-Mer, tamoxifen-inducible Cre recombinase plasmid (see Methods for details), followed by hydroxytamoxifen (4-OHT) treatment (Figure 3A). The efficiency of Cre-recombination was low, and *Dicer*^Δ/Δ ^colonies were quickly overgrown by heterozygous *Dicer*^lox/Δ ^colonies. Nevertheless, we isolated three independent sub-clones, S5, S6 and E5, that had deleted the Dicer RNase III domain on both alleles (Figure 3B).

To ensure that deletion of the RNase III domain completely abolished *Dicer *function we performed Northern blot hybridisation of RNA from floxed and Dicer-deficient clones with a probe for the micro RNA, miR-292 (Figure 3C). The result confirmed the absence of miR-292 and enrichment of the corresponding pre-miRNA in the S5 and S6 *Dicer *mutant clones relative to control floxed cell lines.

We went on to characterise Dicer-deficient ES cell lines. In accordance with previous observations [32], *Dicer*^Δ/Δ ^clones overexpress major satellite repeats (data not shown) and are not capable of differentiation. We attempted to differentiate the cells by withdrawing LIF. In contrast to parental *Dicer*^lox/lox^cells, D3 *Dicer*^Δ/Δ ^clones did not form embryoid bodies, but stayed in irregular-shaped clumps which subsequently attached and continued to grow. After 11 days of differentiation levels of the pluripotent ES cell markers Oct4, Nanog, Fgf4 and Errβ expression levels remained unchanged (Additional file 1). Interestingly, T/Brachyury, which is usually expressed at low levels in wt ES cells, presumably due to a small number of differentiated cells, was completely absent in *Dicer*^Δ/Δ ^clones, and did not appear even after culturing cells for 11 days under differentiation conditions. This result suggests that Dicer-deficient ES cells are either unable to differentiate or alternatively that differentiated cells present in the cultures do not survive.

### Hypomethylation of the *Xist *promoter in Dicer-deficient XY ES cells

To determine whether Dicer deficiency affects *Xist *promoter methylation, we performed MSRE analysis of DNA from control and *Dicer*^Δ/Δ ^clones (Figure 3D and 3E). Interestingly, all *Dicer*^Δ/Δ ^clones demonstrated partial loss of methylation at all of the restriction sites analysed. It should be noted, however, that different clones showed varying degrees of methylation loss, with S5 showing the highest percentage of hypomethylation and S6 the lowest (Figure 3E and 3F).

The cell lines used in this preliminary analysis underwent several rounds of cloning and selection during the derivation process, so we proceeded to derive further lines, in this case making use of ES cells carrying floxed *Dicer *alleles and a tamoxifen inducible Cre recombinase gene targeted into the Rosa26 locus [33]. In this system Cre-recombinase-mediated deletion of the floxed cassette was very efficient following addition of tamoxifen, and we were able to select several individual clones from two different parental *Dicer*^lox/lox ^cell lines, DTCM23 and DTCM49. In addition, we established Dicer-deficient cells from pools of 200–250 colonies of tamoxifen treated cells (Figure 3A). All further analyses were performed in parallel on clones derived by both approaches.

**Figure 4:**
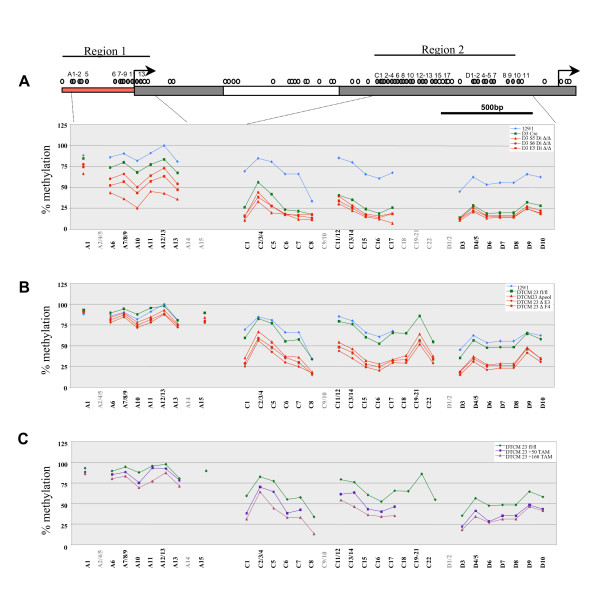
**SEQUENOM mass spectrometry analysis of *Xist *DNA methylation in Dicer-deficient XY embryonic stem cell lines**. Schematic representation of the *Xist *promoter region and 5'end of exon 1 (CpG regions 1 and 2, see Figure 2 for a detailed description). Graphs show the percentage of methylation of specific *Xist *CpG sites in the two groups of *Dicer*^lox/lox ^and deficient embryonic stem (ES) cell lines ((A) and (B)). Average data for at least three independent DNA samples is shown for each CpG site. The wt 129/1 XY ES cell line is included as a reference control on each graph. The dots are joined by lines when consecutive sites were analysed. CpG sites numbered in grey below the graphs indicate that the data points are not available due to low or high fragment mass or due to duplication or overlay of two or more fragments. The average data for two or three CpG sites (for example, A7/8/9) is shown in cases when the sites reside close to each other and could not be resolved as separate fragments. (C) Dynamic of *Xist *CpG island hypomethylation in the DTCM23 floxed cell line exposed to tamoxifen for 50 (blue) or 168 hours (lilac).

To determine *Xist *promoter hypomethylation quantitatively we analysed bisulphite-modified DNA from the D3^lox/lox ^and S5, S6, E5 *Dicer*^Δ/Δ ^clones using SEQUENOM MALDI-TOF mass spectrometry analysis. D3-derived *Dicer*^Δ/Δ ^clones showed substantial hypomethylation both in region 1 and region 2 (Figure 4A). The D3^lox/lox ^parental clone also showed moderate loss of methylation in region 1 and significant loss in region 2, but less than in *Dicer*^Δ/Δ ^clones. The reason for this remains unknown, but could be explained by a mutation or rearrangement that occurred during the several rounds of cell selection to which the D3^lox/lox^ cells were subjected. To exclude the possibility of such an unrelated mutation causing the observed hypomethylation phenotype, we performed mass spectrometry analysis for the DTCM23^lox/lox ^parental cell line and for *Dicer*^Δ/Δ ^clones 23ΔE3, 23ΔF4 and the 23Δpool. The DTCM23^lox/lox ^cell line showed a methylation pattern similar to the 129/1 XY ES control, while all *Dicer*^Δ/Δ ^clones demonstrated hypomethylation most notably in region 2 (Figure 4B). A similar result was obtained for another set of clones, DTCM49^lox/lox ^and *Dicer*^Δ/Δ ^derivatives (Additional file 2A).

To determine the dynamics of methylation loss, we treated the DTCM23^lox/lox ^and DTCM49^lox/lox ^cell lines with 4-OHT and collected DNA for SEQUENOM analysis 50 and 168 hours later. The result shows that *Xist *promoter hypomethylation occurs rapidly following the deletion of *Dicer *and that further hypomethylation occurs with continued cell passaging, again most notably in region 2 (Figure 4C and Additional file 2B).

### *Xist *expression is elevated in Dicer-deficient XY ES cells

**Figure 5:**
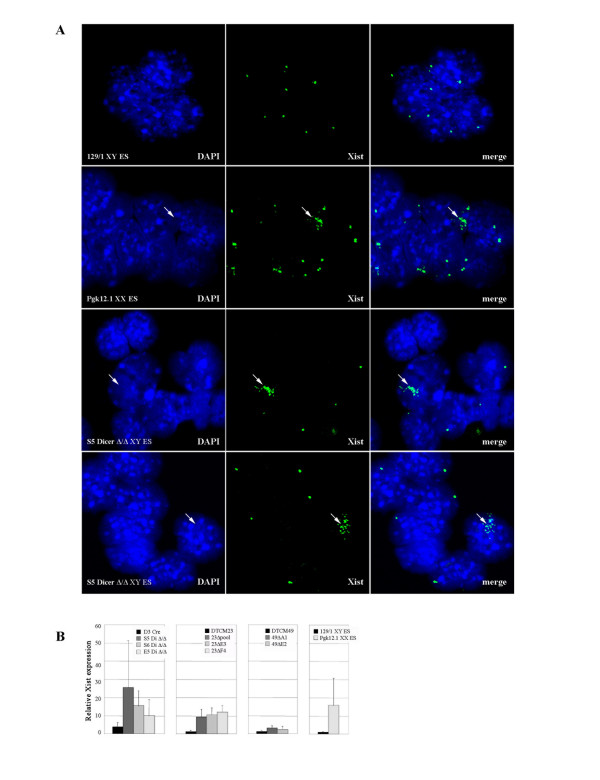
**Analysis of *Xist *expression in Dicer-deficient XY embryonic stem cells**. (A) RNA FISH analysis in the undifferentiated wt XY ES cell line (129/1), wt XX ES cell line (Pgk12.1) and Dicer-deficient XY ES clone (S5) using full length DIG labelled *Xist *probe. The probe is detected with a FITC-coupled antibody (green) and DNA is counterstained with DAPI. Merged colour images are shown in the right-hand panels. The majority of *Dicer *mutant ES cells show one pinpoint signal per cell corresponding to *Xist *and *Tsix *transcripts, similar to the XY control cell line. A proportion of mutant cells demonstrate an elevated level of *Xist *signal (arrow) which either accumulates tightly along the chromosome, similar to XX cells (compare the two middle panels), or shows more dispersed and scattered localisation in the vicinity of the X chromosome (arrow, bottom panel). Occasional accumulation of *Xist *in undifferentiated Pgk12.1 XX ES cultures is attributable to a small proportion of differentiatingcells. (B) Quantitative RT-PCR analysis of *Xist *expression in *Dicer*^lox/lox ^and deficient XY ES cells. The three panels show three groups of *Dicer *null clones with corresponding floxed parental controls. The right-hand panel shows relative level of *Xist *expression in 129/1 XY and Pgk12.1 XX ES cells. All data is normalised to β-actin transcript levels and presented relative to the 129/1 *Xist *RNA level. Accumulation of *Xist *RNA detected by RNA FISH correlates with elevated level of *Xist *transcript determined by quantitative RT-PCR.

To test how hypomethylation affects the transcriptional status of the *Xist *promoter in Dicer-deficient ES cell lines, we performed RNA fluorescent *in situ *hybridisation (FISH) analysis on the S5 *Dicer*^Δ/Δ ^ES clone using probes detecting *Xist *and *Tsix *transcripts. The majority of the S5 cells showed a single pinpoint signal similar to the control 129/1 XY ES cells. However, occasionally there were cells with an upregulated *Xist *signal, which either 'painted' the X chromosome (Figure 5A, third panel from the top) or was scattered in the vicinity of the chromosome (Figure 5A, bottom panel). On average about 10% of cells showed this upregulation pattern, confirming that hypomethylation of the *Xist *promoter compromised regulation of *Xist *expression.

Next we analysed *Xist *expression quantitatively for all *Dicer*^lox/lox ^control and *Dicer*^Δ/Δ ^ES clones. The data for *Xist *expression was normalised to β-actin and then to the level of the *Xist *transcript in control 129/1 XY ES cells and is presented in Figure 5B. All *Dicer*^Δ/Δ ^ES clones showed an elevated level of *Xist *expression in comparison with their corresponding parental floxed cell lines; however, the absolute level of *Xist *upregulation varied between individual clones. It is worth noting that while *Xist *expression in DTCM23^lox/lox ^and DTCM49^lox/lox ^was the same as 129/1 control, the D3^lox/lox ^clone showed elevated expression, consistent with the observed promoter hypomethylation.

### Hypomethylation of the *Xist *promoter in Dicer-deficient XY ES cells correlates with depletion of *de novo *DNA methyltransferases

*Xist *promoter hypomethylation in Dicer-deficient ES cells could be due to a direct effect on recruitment of DNA methyltransferases (Dnmts), for example mediated by the RNAi pathway. Alternatively sense and/or antisense transcription may be required to establish other features of the underlying chromatin structure at the *Xist *promoter, for example specific histone lysine methylation marks, which in turn could have an indirect impact on the recruitment of Dnmts. To test the indirect model we analysed the repressive histone modifications H3K9me2 (data not shown), H3K27me3, H4K20me3 as well as the active mark H3K4me2 over the *Xist *locus in wt and Dicer-deficient ES cells using chromatin immunoprecipitation (ChIP). None of these histone modifications showed a significant change in Dicer-deficient ES cells (Additional files 3 and 4).

**Figure 6:**
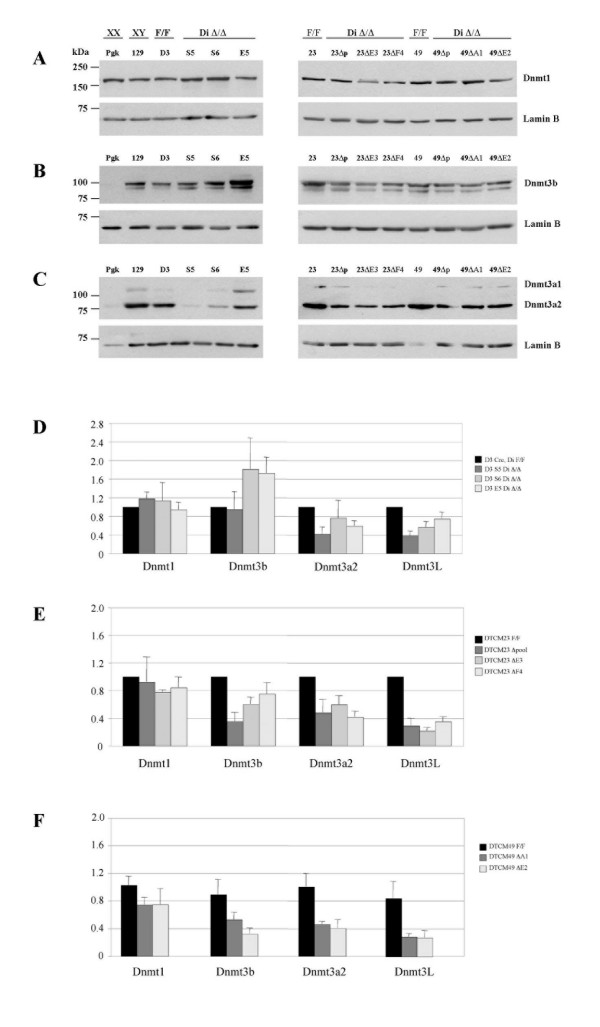
**Analysis of *de novo *DNA methyltransferases in Dicer-deficient XY embryonic stem cell lines**. Western blot analysis of Dnmt1 (A), Dnmt3b (B) and Dnmt3a (C) in Pgk12.1 XX (Pgk), 129/1 (129), *Dicer*^lox/lox ^(F/F) and Dicer-deficient (Di Δ/Δ) XY embryonic stem (ES) cell lines. Lamin B was used as a loading control. Quantitative reverse transcription polymerase chain reaction (RT-PCR) analysis of Dnmt1, Dnmt3b, Dnmt3a2 and Dnmt3L transcription in *Dicer*^lox/lox ^(Di F/F) and Dicer-deficient XY ES cell lines (D)-(F). Two or three primer pairs were used for each Dnmt and average data from triplicate measurements is shown. All data is normalised to Idh2 and β-actin transcript levels and presented relative to D3Cre Dnmt level for S5, S6 and E5 clones and to DTCM23 F/F for DTCM23 and DTCM49 groups of clones.

The absence of detectable changes in histone modification in *Dicer*^Δ/Δ ^cells relative to floxed parental cells suggested that hypomethylation results from a direct effect on recruitment of Dnmts. It was reported previously that *Xist *promoter methylation is mediated by *de novo *DNA methyltransferases Dnmt3a and/or Dnmt3b [34]. We therefore went on to analyse the levels of these enzymes, and also of the maintenance methyltransferase Dnmt1, using Western blotting. In agreement with previous observations Dnmt3a and Dnmt3b levels were very low in XX compared with XY control ES cell lines [31]. Interestingly we also observed reduced levels of Dnmt3a in *Dicer*^Δ/Δ ^clones in comparison with *Dicer*^lox/lox ^controls (Figure 6A–C). The most affected clone was S5, which showed approximately five times less Dnmt3a protein in comparison with the D3 control. The DTCM23 and DTCM49 sets of *Dicer*^Δ/Δ ^clones demonstrated a depletion of Dnmt3a and also a slight decrease in Dnmt3b levels.

To determine whether Dnmt3a/b depletion resulted from transcriptional or post-transcriptional regulation, we performed quantitative RT-PCR analysis with primers designed for Dnmt1, Dnmt3a2 (the major Dnmt3a isoform in ES cells), Dnmt3b (for all Dnmt3b isoforms) and Dnmt3L (Figure 6D–F). Consistent with the Western data, we found that levels of Dnmt3a2 transcript are consistently lower in *Dicer*^Δ/Δ ^clones than in controls. We also found that the level of Dnmt3L, a functional partner of Dnmt3a2, is much reduced in Dicer-deficient clones. The level of Dnmt3b was reduced in the DTCM series of clones, but not in the S5, S6 and E5 *Dicer*^Δ/Δ ^clones, in accordance with the Western blot results. The level of Dnmt1 was not significantly different between control and Dicer-deficient clones. Affymetrix microarray analysis of RNA from D3^lox/lox ^versus S5 *Dicer*^Δ/Δ ^clones also showed a 2.4-fold decrease of Dnmt3a and 3.3-fold decrease of Dnmt3L (data not shown).

To determine whether hypomethylation occurs at other loci in *Dicer*^Δ/Δ ^clones we analysed methylation at the differentially methylated regions (DMRs) of two imprinted genes, H19 and Igf2rAir (Additional file 5). In both examples we observed hypomethylation specifically in *Dicer*^Δ/Δ ^clones. It should be noted that two recent studies reported hypomethylation of repetitive and unique sequences in *Dicer*^Δ/Δ ^ES cells and attributed this to reduced levels of Dnmts [35,36].

Given the requirement for Dnmt3a/b in *Xist *promoter methylation [31,34,37], we conclude that hypomethylation in *Dicer*^Δ/Δ ^clones is most likely attributable to decreased levels of expression of these enzymes rather than deficiency of a dsRNA-mediated transcriptional gene silencing mechanism.

### Monoallelic *Xist *expression and *Xist *RNA spreading in Dicer-deficient XX embryos

**Figure 7:**
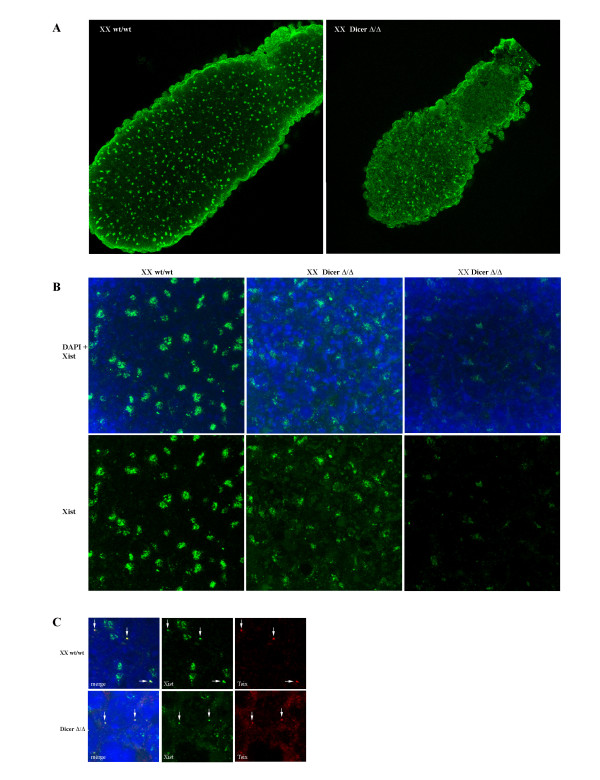
**RNA fluorescent *in situ *hybridisation analysis of *Xist*/*Tsix *expression in *Dicer*^Δ/Δ ^embryos at E6.5**. (A) RNA FISH analysis of *Xist *expression in representative whole mount E6.5 wt and Dicer-deficient embryos using a full-length DIG labelled *Xist *probe. The probe is detected with a FITC-coupled antibody (green). Examples show combined confocal optical sections through the whole embryo (15 sections with a distance 0.35 μm between each section were merged for each embryo; 63× objective). (B) Enlarged view (×3) of the epiblast part of the whole mount E6.5 wt and Dicer-deficient embryos after RNA FISH with *Xist *probe shown in (A). The Xist probe is detected with a FITC-coupled antibody (green) and DNA is counterstained with DAPI. (C) RNA FISH analysis of *Xist *(green) and *Tsix *(red) expression in whole mount E6.5 wt and Dicer-deficient embryos. Examples show combined confocal optical sections through the epiblast part of the embryo (10 sections with a distance 0.35 μm between each section were merged for each embryo). Pinpoint *Xist*/*Tsix *signal (arrow) is visible in wt and in *Dicer*^Δ/Δ ^embryos.

Finally, we wished to test for a role for Dicer in the initiation of random X inactivation in XX cells and also to determine whether the RNAi pathway is important in spreading *Xist *RNA on the inactive X chromosome. As Dicer-deficient ES cells are not capable of differentiation and we have not been able to isolate a stable Dicer-deficient XX ES cell line, we analysed Dicer-deficient XX embryos produced by mating *Dicer*^wt/Δ ^heterozygous mice. In general, *Dicer*^Δ/Δ ^embryos survived until approximately E7.5–E8.5 and were smaller than their heterozygous or wt littermates, consistent with a study published previously [38]. This afforded the opportunity to analyse the initiation of random X inactivation that commences at approximately E5.5. We carried out whole mount RNA FISH using *Xist *and *Tsix *probes on E6.5 embryos (Figure 7). The *Dicer*^Δ/Δ ^XY embryos showed a *Xist*/*Tsix *pinpoint signal similar to their wt and heterozygote XY littermates, while the *Dicer*^Δ/Δ ^female embryos had both pinpoint and accumulated *Xist *transcripts, suggesting that Dicer is not affecting the initiation step of random X inactivation in the inner cell mass (ICM). The presence of *Xist *clouds in cells of XX embryos implies that spreading of *Xist *RNA also does not require Dicer activity. We did note that XX embryos showed weaker and more disrupted *Xist *signal with stronger general background for both *Xist *and *Tsix *probes, and that this varied from embryo to embryo. This is likely due to the onset of embryo lethality and apoptosis in mutant embryos. Overall these observations further support our conclusion that X inactivation can occur independent of the RNAi pathway.

## Discussion

In this study we set out to further explore the mechanism of regulation of *Xist *gene expression at the onset of random X inactivation. In particular, we wished to understand the importance of sense and antisense transcription in *Xist *promoter repression and the possible involvement of the RNAi pathway. We demonstrate that enhanced sense transcription in ES cells prior to the onset of X inactivation reduces the levels of DNA methylation at the *Xist *promoter. In addition, we found that antisense *Tsix *transcription is required for *Xist *promoter methylation in undifferentiated ES cells. DNA hypomethylation of the *Xist *promoter was also observed in Dicer-deficient ES cells but further analysis indicated this is an indirect effect attributable to reduced levels of the *de novo *methyltransferases Dnmt3a, Dnmt3b and Dnmt3L. Similarly, Dicer-deficient embryos displayed normal allelic regulation of *Xist*/*Tsix *expression and, moreover, *Xist *RNA spreading occurred on a single X chromosome in Dicer-deficient XX embryos. We conclude that *Tsix*-mediated regulation of *Xist *expression and other steps in the X inactivation process occur independent of the RNAi pathway.

### The role of sense and antisense transcription in regulating *Xist *promoter methylation

Previously we demonstrated that modified *Xist *alleles showing increased sense transcription from heterologous promoters in ES cells also show preferential X inactivation in XX heterozygous animals [12,13]. Here we have extended this finding, showing that these modified *Xist *alleles are partially hypomethylated over the *Xist *promoter region, providing a mechanistic basis for preferential expression in XX heterozygotes. Similarly the mutant *Xist *allele in XT67E1 XX ES cells has ectopic transcription in the sense direction and complete hypomethylation of promoter CpG sites that lie immediately upstream of the deleted region. In this case hypomethylation cannot be correlated with increased probability of expression because the deleted allele is lacking the *Xist *TSS.

Enhanced sense transcription antagonises *Xist *promoter methylation even when normal levels of antisense *Tsix *RNA are present. However, our data show that *Tsix *transcription is important for the establishment of *Xist *promoter methylation in ES cells, that is, prior to X inactivation. This contrasts with a previous report in which a different *Tsix *mutant allele was suggested to have a role in *Xist *promoter methylation during ES cell differentiation, but not prior to onset of random X inactivation in undifferentiated ES cells [6]. This discrepancy is explained in part by the fact that hypomethylation occurs more in region 2 than in region 1 (this study), and Sun et al. [6] analysed only region 1. A second possible factor is that independent *Tsix *mutant XY ES cells behave differently. Notably the Δ65, 2 lox, pAA2Δ1.7 and Δ34# 1 XY ES cell lines all upregulate *Xist *inappropriately upon differentiation [7,9,11,16], presumably at least in part due to promoter hypomethylation, whereas the ΔCpG cell line maintains *Xist *repression throughout differentiation [8]. It is possible that this difference arises because the secondary pathway, *Xist *repression linked to the pluripotency program [18], plays a more dominant role in the ΔCpG XY ES cell line.

### Dicer indirectly regulates *Xist *promoter methylation in ES cells

We wanted to determine whether regulation of *Xist *promoter methylation by sense and antisense RNAs was mediated by the RNAi pathway. We found that Dicer-deficient ES cells show *Xist *promoter hypomethylation and moderate upregulation of *Xist *transcripts, an effect that was observed in a number of independent cell lines, albeit with some variation in degree. However a number of facts lead us to conclude that this is an indirect consequence of *Dicer *deletion. First and foremost we observed downregulaton of the *de novo *DNA methyltransferases, Dnmt3a, Dnmt3b and Dnmt3L in Dicer-deficient cells. Several studies have demonstrated that Dnmt3a/3b levels are important for maintaining *Xist *promoter methylation [31,34,37], indicating that reduced Dnmt levels are sufficient to account for *Xist *promoter hypomethylation in Dicer-deficient cells. Consistent with this idea we observed hypomethylation at imprinted loci, and two recent studies have reported hypomethylation of subtelomeric repeats [35] and promoters of *Oct4, Tsp50 *and *Sox30 *genes [36] in independently isolated Dicer-deficient cell lines. Importantly the hypomethylation phenotype seen in these studies was complemented by ectopic expression of Dnmt transgenes, indicating that the RNAi pathway is not directly involved. These studies also demonstrated that downregulation of Dnmts results from overexpression of Rbl2 that is, in turn, normally subject to negative regulation by the miR-290 cluster miRNAs. In agreement with this conclusion we also found that the level of Rbl2 transcript is upregulated 4.4-fold in the Dicer-deficient cell lines described here (data not shown).

A second line of evidence that argues that the RNAi pathway is not required for *Xist *gene regulation and random X inactivation comes from our analysis of Dicer-deficient embryos at early post-implantation stages. Here we observed appropriate *Xist *and *Tsix *expression patterns in XY and upregulation of *Xist *from a single allele in XX embryos. The fact that XX embryos show reduced intensity of staining of *Xist *domains most likely reflects that embryo lethality occurs shortly after the stage we examined, E6.5 [38]. It should be noted that we cannot formally rule out that in XX embryos the pattern we observe represents persistence of the imprinted X inactivation pattern, that is, that Dicer deficiency results in failure to erase imprinted X inactivation prior to establishing random X inactivation.

### Regulation of *Xist *promoter methylation

Given the evidence that the RNAi pathway does not mediate *Xist *promoter regulation through sense and antisense transcription, what are the alternative mechanisms? The fact that we observe hypomethylation in undifferentiated *Tsix*-deficient XY ES cells suggests a direct link between antisense transcription and promoter CpG methylation. That some CpG methylation is retained in the *Tsix *mutant cells may indicate a redundant mechanism for recruiting DNA methylation to the promoter, for example, relating to *Xist *repression by the pluripotency program [18,20], or alternatively may simply reflect that the maintenance methyltransferase activity, Dnmt1, is sufficient to retain promoter methylation up to a defined level.

Assuming that *Xist *promoter methylation prior to the onset of X inactivation is indeed a consequence of antisense *Tsix *expression we can envisage two possible mechanisms. Either *Tsix *directly recruits *de novo *Dnmts, Dnmt3a and Dnmt3b, as has been suggested previously for Dnmt3a [6], or alternatively *Tsix *may mediate other chromatin changes at the *Xist *promoter, for example reducing H3K4 methylation as suggested previously [3], DNA hypomethylation being a secondary consequence. Whilst we are unable to distinguish between these possibilities at present, it is interesting to note that H3K4 methylation antagonises the binding of Dnmt3a/Dnmt3L dimers to nucleosome [39], providing a possible mechanism for hypomethylation of DNA driven by a *Tsix*-mediated reduction in H3K4 methylation levels. In the context of this model increased sense transcription from upstream heterologous or cryptic promoters may antagonise *Xist *promoter methylation by increasing the H3K4 methylation levels locally.

## Conclusion

We have shown that sense and antisense transcription over the *Xist *promoter can modulate DNA methylation levels prior to the onset of random X inactivation, providing a mechanistic basis for skewed X inactivation patterns in mutants that alter sense or antisense RNA levels. We went on to investigate the possible involvement of the RNAi pathway. Our analysis of Dicer-deficient ES cells demonstrated *Xist *promoter hypomethylation, but this appears to be a secondary consequence of reduced levels of Dnmts. Consistent with this, appropriate *Xist/Tsix *expression patterns were seen to occur in Dicer-null embryos. Based on these observations we conclude that the RNAi pathway is probably not required for X chromosome inactivation in mammals.

## Methods

### ES cell line derivation and maintenance

*Dicer*^lox/lox ^ES cell lines were derived from the ICM of E3.5 embryos using two approaches. In the first approach, ES cell lines were derived from mice homozygous for a *Dicer*^lox ^allele. The established *Dicer*^lox/lox ^XY ES cell line D41 was subsequently lipofected with pCAG-Mer-Cre-Mer plasmid, carrying tamoxifen inducible Cre-recombinase. Clone D41D3Cre (D3Cre) was treated with 800 nM 4-hydroxytamoxifen (4-OHT, Sigma), plated at clonal density, and individual colonies were picked, expanded and then tested by genomic PCR for the loss of the Dicer RNase III domain. Three clones S5, S6 and E5 were identified that showed loss of the Dicer RNase III floxed cassette.

In the second approach ES cell lines were derived from mice homozygous for a *Dicer*^lox ^allele crossed to animals either homozygous or heterozygous for tamoxifen inducible Cre-recombinase targeted into the Rosa26 locus (obtained from Artemis Pharmaceuticals; [33]). Two derived parental XY ES cell lines, DTCM23 and DTCM49, were treated with 800 nM 4-OHT and plated at clonal density. Individual clones as well as pools of approximately 200–250 clones were genotyped for the loss of the RNase III floxed cassette. Dicer-deficient clones DTCM23 ΔE3, ΔF4, DTCM49 ΔA1 and ΔE2 as well as a pool of Dicer-deficient clones DTCM23 Δpool were selected for further analysis.

ES cells lines were derived and maintained on a feeder layer (mitomycin-inactivated primary mouse embryonic fibroblasts) in Dulbecco's Modified Eagle Medium (DMEM) supplemented with 10% foetal calf serum (FCS, Autogen Bioclear), 7% Knockout Serum Replacement (KSR), 2 mM L-glutamine, 1× non-essential amino acids, 50 μM 2-mercaptoethanol, 50 μg/ml penicillin/streptomycin (all from Invitrogen) and LIF-conditioned medium, made in house, at a concentration equivalent to 1000 U/ml. Cells were grown at 37°C in a humid atmosphere with 5% CO_2_.

### Methylation-sensitive restriction enzyme analysis

*Dicer*^lox/lox ^and deficient ES cells were pre-plated for 30 minutes to minimise feeder cell contamination and then grown for 2–3 days until confluent on plates coated with 0.1% gelatin. Genomic DNA was phenol/chloroform extracted by the standard procedure. The genotype of each preparation was confirmed by PCR using primers SEQ28290 (agtaatgtgagcaatagtcccag), Di31831 (agtgtagccttagccatttgc) and Di32050AS (ctggtggcttgaggacaagac) and the following PCR conditions: 95°C for 4 minutes; (95°C for 30 seconds; 60°C for 30 seconds; 72°C for 30 seconds) × 35 cycles. PCR fragments were resolved on a 2.5% agarose gel, giving a 259 bp fragment for the wt allele, a 390 bp fragment for the floxed allele and a 309 bp fragment for the *Dicer *null allele (see Figure 3B).

Genomic DNA was digested to completion with either EcoRI or BamHI restriction enzymes according to the manufacturer's instructions, ethanol precipitated and re-dissolved in TE buffer (10 mM Tris, pH 8.0; 1 mM EDTA). Using methylation-sensitive enzymes, 10 μg DNA aliquots were re-digested, separated by electrophoresis on a 1% agarose gel and blotted onto a GeneScreen nylon filter (Perkin Elmer Life Sciences). Hybridisation with *Xist *probe 3 (from -37 bp to +952 bp relative to *Xist *start site P_1_) was performed as described previously [40]. Images were collected on a PhosphorImager instrument (Molecular Dynamics) and fragment intensity quantification was performed using ImageQuant software (Molecular Dynamics).

### SEQUENOM methylation analysis

Genomic DNA was extracted in the same way as for methylation-sensitive Southern analysis. We bisulphite treated 2 μg aliquots of high molecular weight DNA using the EZ DNA methylation kit (Zymo Research). Treatment was performed essentially according to the manufacturer's instructions with a modification in the conversion step, that included 20 cycles of sample treatment with the following conditions (95°C for 30 seconds; 50°C for 15 minutes). Converted DNA was purified on the columns and eluted in 100 μl of water. We used 5 μl of the sample per 25 μl PCR reaction.

HotStarTaq DNA Polymerase kit (Qiagen) was used to amplify modified DNA and PCR primers and the conditions used are described in the Table 1. PCR fragments were sent to SEQUENOM GmbH company (Hamburg, Germany) for *in vitro *transcription and subsequent MALDI-TOF mass spectrometry analysis using EpiTYPER software [30].

**Table 1:** Primers and polymerase chain reaction (PCR) conditions for bisulphite methylation analysis

Region/gene	Primer, forward	Primer, reverse	PCR conditions
*Xist*, region 1A – 1st round	MM3, gttaattaatgtagaagaatttttagtgttta	MM2, aaatattcccccaaaactccttaaataa	94°C for 15 minutes; (94°C for 20 seconds; 54°C for 30 seconds; 72°C for 90 seconds) × 25 cycles; 72°C for 5 minutes
*Xist*, region 1A – nested	Tag 6F, aggaagagagttgtaaaataggataattttttattat	T7 6R, cagtaatacgactcactatagggagaaggctatattcccccaaaactccttaaata	94°C for 15 minutes; (94°C for 20 seconds; 54°C for 30 seconds; 72°C for 30 seconds) × 5 cycles; (94°C for 20 seconds; 60°C for 30 seconds; 72°C for 30 seconds) × 25 cycles; 72°C for 5 minutes
*Xist*, region 2C	Tag 1F, aggaagagagattttgtttttaaattgagtgggtg	T7 1R, cagtaatacgactcactatagggagaaggcttaaaccctatcccctaatcctctac	94°C for 15 minutes; (94°C for 20 seconds; 60°C for 30 seconds; 72°C for 1 minute) × 5 cycles; (94°C for 20 seconds; 68°C for 30 minutes; 72°C for 1 minute) × 40 cycles; 72°C for 5 minutes
*Xist*, region 2D	Tag 5F, aggaagagagagggtgtgtgtgtatatggattttt	T7 MM5, cagtaatacgactcactatagggagaaggcttaactcaaacataaaactaacatataacaca	94°C for 15 minutes; (94°C for 20 seconds; 54°C for 30 seconds; 72°C for 1 minute) × 5 cycles; (94°C for 20 seconds; 58°C for 30 seconds; 72°C for 1 minute) × 40 cycles; 72°C for 5 minutes
Igf2r	Tag Igf2r_F2, aggaagagaggtgtggtatttttatgtatagttagg	T7 Igf2r_R cagtaatacgactcactatagggagaaggctaaatatcctaaaaatacaaactacac	94°C for 15 minutes; (94°C for 20 seconds; 54°C for 30 seconds; 72°C for 1 minute) × 45 cycles; 72°C for 5 minutes
H19A	Tag H19A, aggaagagaggagaaaatagttattgtttatagtttt	T7 H19A, cagtaatacgactcactatagggagaaggctcctaaaatactaaacttaaataacccacaa	94°C for 15 minutes; (94°C for 20 seconds; 50°C for 30 seconds; 72°C for 1 minute) × 45 cycles; 72°C for 5 minutes
H19B	Tag H19A, aggaagagagttagtgtggtttattataggaaggtatagaagt	T7 H19A, cagtaatacgactcactatagggagaaggct taaacctaaaatactcaaactttatcacaac	94°C for 15 minutes; (94°C for 20 seconds; 50°C for 30 seconds; 72°C for 1 minute) × 5 cycles; (94°C for 20 seconds; 58°C for 30 seconds; 72°C for 1 minute) × 40 cycles; 72°C for 5 minutes

### RT-PCR analysis

RNA was isolated from ES cells using TRIzol reagent (Sigma) according to the manufacturer's instructions. RNA was routinely treated with Turbo DNA-free reagent (Ambion) to exclude the possibility of DNA contamination. cDNA synthesis was primed from random hexamers (GE Healthcare) with Superscript III reverse transcriptase (Invitrogen). Strand-specific RT-PCR for *Xist *amplicons 4, 5, 51 and 51mut was performed according to the method described previously [13]. Primers and PCR conditions are given in Table 2.

**Table 2:** Primers and polymerase chain reaction (PCR) conditions for quantitative reverse transcription PCR

Region/gene	Primer, forward	Primer, reverse	PCR conditions
*Xist*, Amp 4	TN4s ttctaccctttcctctcctcatc	JTL4as, gaggtacgtaagctcagtga	95°C for 5 minutes; (95°C for 30 seconds; 60°C for 30 seconds; 72°C for 30 seconds) × 40 cycles
*Xist*, Amp 5	Qmex4, gcaaggaagacaaaggctcaaagaat	Qmex52 ggagagagaaccaaatagagcagaat	95°C for 3 minutes; (95°C for 20 seconds; 61°C for 15 seconds; 72°C for 30 seconds) × 40 cycles
*Xist*, Amp 51	DE-SOL2, tgcaatctttgtggccactcctcttctg	TN51, tatcaaaacgtcaaaaatctcg	95°C for 5 minutes; (95°C for 30 seconds; 60°C for 30 seconds; 72°C for 30 seconds) × 40 cycles
*Xist *mut, Amp 51m	DE-SOL2, tgcaatctttgtggccactcctcttctg	neoTN9, catcgcattgtctgagtaggtgtc	95°C for 5 minutes; (95°C for 30 seconds; 64°C for 30 seconds; 72°C for 30 seconds) × 40 cycles
Dnmt1	Dnmt1_F1, agatccactgtggcaagaaga	Dnmt1_R1, ctgaagttcaccacagcttcc	95°C for 3 minutes; (95°C for 20 seconds; 60°C for 20 seconds) × 40 cycles
Dnmt1	Dnmt1_F2, agggaccatatctgcaaggac	Dnmt1_R2, gctgctgtagccatttttcac	95°C for 3 minutes; (95°C for 20 seconds; 60°C for 20 seconds) × 40 cycles
Dnmt3a2	Dnmt3a2_F1, cagacgggcagctatttacag	Dnmt3a2_R1, ggttctcttccacagcattca	95°C for 3 minutes; (95°C for 20 seconds; 60°C for 20 seconds) × 40 cycles
Dnmt3a2	Dnmt3a2_F2, ggctcacacctgagctgtact	Dnmt3a2_R2, cctcctccaccttctgagact	95°C for 3 minutes; (95°C for 20 seconds; 60°C for 20 seconds) × 40 cycles
Dnmt3b	Dnmt3b_F1, caagcgcctcaagacaaatag	Dnmt3B_R1, gcgatcccggcaactctgaca	95°C for 3 minutes; (95°C for 20 seconds; 60°C for 20 seconds) × 40 cycles
Dnmt3b	Dnmt3b_F2, cgagaacaaaagtcgaagacg	Dnmt3b_R2, gggttcttctttccacaggac	95°C for 3 minutes; (95°C for 20 seconds; 60°C for 20 seconds) × 40 cycles
Dnmt3b	Dnmt3b_F3, ccattcttctggatgttcgag	Dnmt3b_R3, tctgatggagttcgacttggt	95°C for 3 minutes; (95°C for 20 seconds; 60°C for 20 seconds) × 40 cycles
Dnmt3L	Dnmt3L_F1, cttgtttgagggagggttatg	Dnmt3L_R1, gtacagtcggggctctcacag	95°C for 3 minutes; (95°C for 20 seconds; 60°C for 20 seconds) × 40 cycles
Dnmt3L	Dnmt3L_F2, agacaactacccgcttccttc	Dnmt3L_R2, ctcttcttcctttggggtcag	95°C for 3 minutes; (95°C for 20 seconds; 60°C for 20 seconds) × 40 cycles
Dnmt3L	Dnmt3L_F3, cccctaggcagctcttgtgat	Dnmt3L_R3, gcgggtagttgtctcttggtc	95°C for 3 minutes; (95°C for 20 seconds; 60°C for 20 seconds) × 40 cycles
Idh2	Idh1F, agaaaatgtggaagagccctaacg	Idh1R, tgccagctcgatctaccacaaaat	95°C for 3 minutes; (95°C for 20 seconds; 60°C for 20 seconds) × 40 cycles
β-actin	BA11, gatatcgctgcgctggtcgt	BA2, agatcttctccatgtcgtcc	95°C for 3 minutes; (95°C for 20 seconds; 60°C for 20 seconds) × 40 cycles

Real-time PCR was performed with SYBR Green PCR Master Mix (Bio-Rad) on a Chromo4 Real-time PCR System (Bio-Rad). PCR primers and conditions are listed in Table 2. A melting curve test was performed at the end of each experiment to ensure the specificity of amplification. The data was normalised to β-actin and Idh2 and then to one of the control samples in the set. Each amplicon was analysed at least twice in triplicate on independent cDNA preparations.

### Northern blot analysis

Total RNA (20–30 μg), isolated using TRIzol reagent, was separated by PAGE on a 15% urea-containing gel. RNA was transferred to Hybond-XL nylon membrane using a Bio-Rad semi-dry blot apparatus at a constant current of 2.1 mA/cm^2 ^for 1 hour. The membrane was UV-crosslinked with 1000 μJ in a Stratagene UV crosslinker and hybridised with ^32^P-dCTP labelled mi292as probe. The image was acquired on a PhosphorImager instrument.

### RNA FISH analysis

RNA FISH was performed as described previously [7,41]. p*Xist*, an 18 kb DNA fragment spanning the whole *Xist *transcript, was labelled using digoxygenin-16-dUTP nick translation mix (Roche) and detected with antidigoxygenin fluorescein isothiocyanate (AD-FITC) antibody raised in sheep (Roche), followed by anti-sheep fluorescein isothiocyanate (FITC) antibody (Vector Laboratories). Images were acquired on a Leica TCS SP5 confocal microscope using LAS AF software.

For whole mount RNA FISH E6.5 embryos were obtained from crosses between mice heterozygous for the *Dicer *RNase III domain deletion. Embryos were dissected from uteri, rinsed in pre-chilled phosphate buffered saline (PBS), and permeabilised in Cytoskeletal (CSK) buffer for 10 minutes on ice. The washes were carried out in 3 cm Petri dishes kept on ice throughout the whole procedure. The embryos were fixed in 4% formaldehyde for 15 minutes on ice and rinsed in pre-chilled PBS. All aforementioned solutions included 0.1% Tween-20 (Sigma) to prevent the embryos from sticking. Embryos were then dehydrated through an ethanol series (70%, 80%, 90%, 100%). The procedure was performed on a glass slide with a depression (VWR). After the last dehydration wash, ethanol was allowed to evaporate and 15 μl of hybridisation solution, containing DIG-labelled *Xist *and biotinylated *Tsix *probes, were added immediately. The slide was covered with a coverslip, sealed with rubber cement, and the hybridisation was performed overnight at 37°C. The *Xist *probe was full-length *Xist *cDNA and the *Tsix *probe was a 4.6 kb EcoRI fragment surrounding *Tsix *major start site. The post-hybridisation washes were as described previously [41] with a modification, which included addition of 0.1% Tween-20 to all solutions. The *Xist *probe was detected with AD-FITC antibody raised in sheep (Roche), followed by anti-sheep FITC antibody (Vector Laboratories), and *Tsix *probe was detected with avidin-Texas red (AV-TR) followed by biotinylated anti-avidin antibody and then again with AV-TR. All antibodies were from Vector Laboratories unless otherwise stated. Images were acquired on a Leica TCS SP5 confocal microscope using LAS AF software. After imaging, each embryo was genotyped by PCR to determine sex and *Dicer *genotype.

### Western analysis

Western blot analysis was performed as described previously [31] with some modifications. Briefly, proteins were separated on 8% SDS-PAGE gels and transferred in 1× Transfer buffer (48 mM Tris, 39 mM Glycine, 0.037% SDS, 20% methanol) at 100 mA/gel for 45 minutes, using Bio-Rad semi-dry blotting apparatus. Dnmt3a antibody (working dilution 1:250) and Dnmt3b antibody (WD 1:300) were from Alexa Biosciences; Dnmt1 antibody (WD 1:250) was from Abcam; and LaminB antibody (WD 1:2000) was from Santa Cruz. Enhanced chemiluminescence detection was performed as recommended by the manufacturer (GE Healthcare).

All mouse work was carried out in accordance with the United Kingdom's Home Office regulations under the Animals (Scientific Procedures) Act 1986.

## Abbreviations

4-OHT, hydroxytamoxifen; AD-FITC, antidigoxygenin fluorescein isothiocyanate; AV-TR, avidin-Texas red; ChIP, chromatin immunoprecipitation; CSK, Cytoskeletal; DIG, digoxygenin-16-dUTP; DMEM, Dulbecco's Modified Eagle Medium; DMR, differentially methylated region; Dnmt, DNA methyltransferase; ES, embryonic stem; FCS, foetal calf serum; FISH, fluorescent *in situ *hybridisation; FITC, fluorescein isothiocyanate; KSR, Knockout Serum Replacement; ICM, inner cell mass; MALDI-TOF, matrix-assisted laser desorption/ionisation time of flight; MSRE, methylation sensitive restriction enzyme site; PBS, phosphate buffered saline; PCR, polymerase chain reaction; RNAi, RNA interference; RT, reverse transcription; TSS, transcriptional start site; WD, working dilution; wt, wild type; *Xist*, X inactive specific transcript.

## Competing interests

The authors declare that they have no competing interests

## Authors' contributions

TBN, MM and NB conceived of and designed the experiments. TBN, BCP, BSC, SN, CES and YAT performed the experiments. TBN, BCP, CES, YAT and NB analysed the data. BSC, TSp, TAR, TSa and MM contributed reagents/materials. TBN and NB wrote the paper. All authors read and approved the final manuscript.

## Supplementary Material

Additional file 1 Dicer deficient ES cells are incapable of differentiation. Expression of lineage specific markers in *Dicer*^lox/lox ^(D3) and *Dicer *null (S5, S6) undifferentiated cells and cells grown in the absence of LIF for 11 days. ES, trophoblast stem (TS), extraembryonic endoderm (XEN) and somatic (fib) cell lines are included as controls. Note no change in marker expression in *Dicer *null cells after culturing in differentiating conditions for 11 days. Click here for file

Additional file 2 SEQUENOM mass spectrometry analysis of *Xist *DNA methylation in Dicer deficient XY ES cell lines. Schematic representation of the *Xist *promoter region and the 5'end of exon 1 (CpG regions 1 and 2, see Fig. 2 legend for detailed description). The graphs show the percentage of methylation of specific *Xist *CpG sites in DTCM49 *Dicer*^lox/lox ^and deficient ES cell lines (A). Average data for at least three independent DNA samples is shown for each CpG site. The wt 129/1 XY ES cell line is included as a reference control on each graph. The dots are joined by lines when consecutive sites were analysed. CpG sites numbered in grey below the graphs indicate that the data points are not available due to low or high fragment mass or due to duplication or overlay of two or more fragments. The average data for two or three CpG sites (e.g. A7/8/9) is shown in cases when the sites reside close to each other and could not be resolved as separate fragments. (B) Dynamic of *Xist *CpG island hypomethylation in DTCM49 floxed cell line exposed to tamoxifen for 50 hrs (blue) or 168 hrs (lilac). Click here for file

Additional file 3 ChIP analysis of histone modifications across the *Xist*/*Tsix *locus in *Dicer*^lox/lox ^and *Dicer *deficient XY ES cell lines. (A) Schematic representation of the *Xist*/*Tsix *locus. *Xist *exons are shown as black rectangles, *Tsix *exons 2–4 are shown as light grey rectangles. The start sites and the direction of transcription for *Xist*, *Tsix *and *Enox *are shown by arrows. The dark grey boxes underneath the schematic show the position of primers used for ChIP (for primer information see (Navarro et al. 2005)). (B-D) ChIP analysis of histone modifications H3K4me2 (B), H4K20me3 (C) and H3K27me3 (D) across *Xist*/*Tsix *locus in *Dicer*^lox/lox ^(D3Cre) and *Dicer *deficient (S5, S6, E5) ES cell lines. Average data from three independent ChIP experiments is presented as percentage of input. Click here for file

Additional file 4 Supplementary methods: Chromatin Immunoprecipitation. File includes Supplementary method for Chromatin Immunoprecipitation, control primers and PCR conditions used for ChIP analysis and supplementary references. Click here for file

Additional file 5 SEQUENOM mass spectrometry analysis of DNA methylation of imprinted genes in *Dicer *deficient XY ES cell lines. Graphs show the methylation level of specific CpG sites of H19 DMR and Igf2rAir DMR in controls (A) (XY and XX ES cell lines and XX somatic cells) and three groups of *Dicer*^lox/lox ^and deficient ES cell lines (B-D). Average data for at least three independent DNA samples is shown for each CpG site. The wt 129/1 XY ES cell line is included as a reference control on each graph. Dots are joined by lines when consecutive sites were analysed. Grey site numbers below the graphs indicate that the data points are not available due to low or high fragment mass or due to duplication or overlay of two or more fragments. The average data for two or three CpG sites (e.g. A6/7) is shown in cases when the sites reside close to each other and could not be resolved to separate fragments. Click here for file
