# Supplementary figures and images for: Dicer regulates Xist promoter methylation in ES cells indirectly through transcriptional control of Dnmt3a
Source: Epigenetics Chromatin. 2008 Oct 27;1:2. doi: 10.1186/1756-8935-1-2 (PMC2577046; doi:10.1186/1756-8935-1-2)

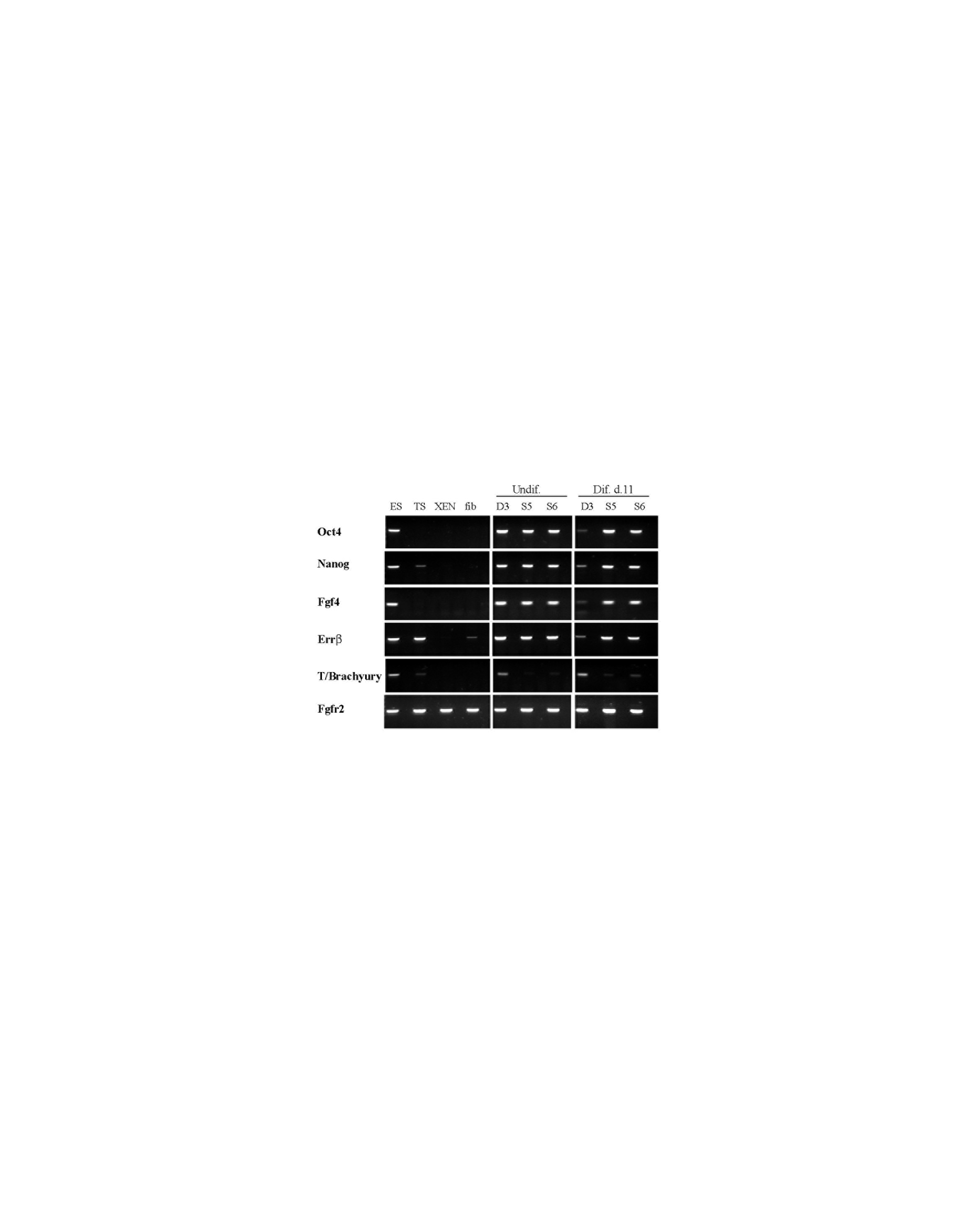

Supplement: Additional file 1 — Dicer deficient ES cells are incapable of differentiation. Expression of lineage specific markers in Dicerlox/lox (D3) and Dicer null (S5, S6) undifferentiated cells and cells grown in the absence of LIF for 11 days. ES, trophoblast stem (TS), extraembryonic endoderm (XEN) and somatic (fib) cell lines are included as controls. Note no change in marker expression in Dicer null cells after culturing in differentiating conditions for 11 days. [file 1756-8935-1-2-S1.jpeg]

A

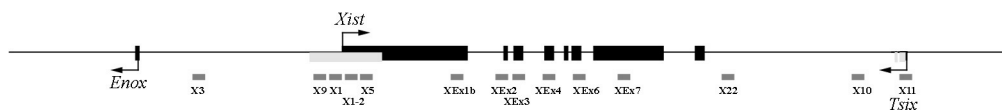

B

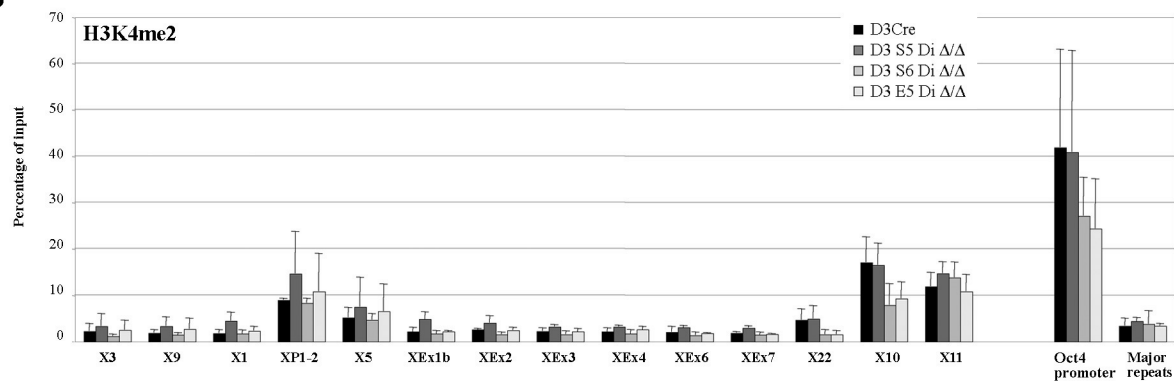

C

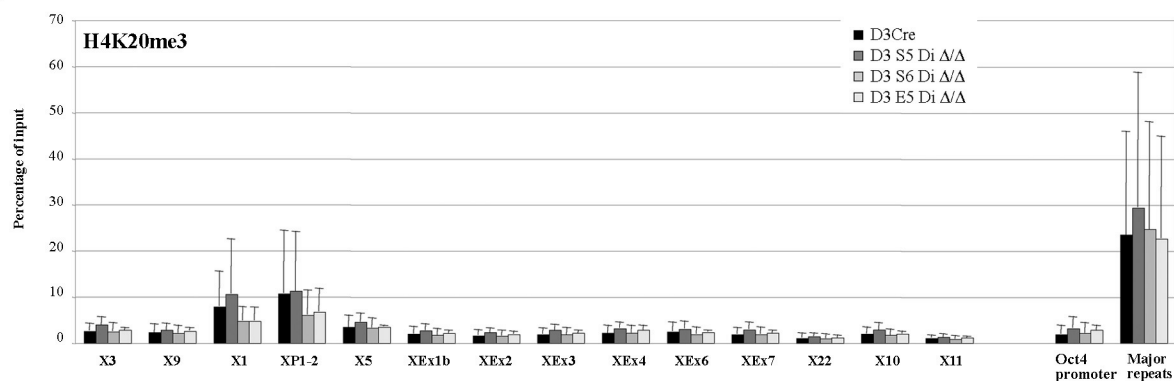

D

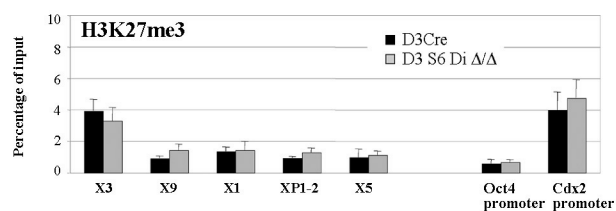

Supplement: Additional file 3 — ChIP analysis of histone modifications across the Xist/Tsix locus in Dicerlox/lox and Dicer deficient XY ES cell lines. (A) Schematic representation of the Xist/Tsix locus. Xist exons are shown as black rectangles, Tsix exons 2–4 are shown as light grey rectangles. The start sites and the direction of transcription for Xist, Tsix and Enox are shown by arrows. The dark grey boxes underneath the schematic show the position of primers used for ChIP (for primer information see (Navarro et al. 2005)). (B-D) ChIP analysis of histone modifications H3K4me2 (B), H4K20me3 (C) and H3K27me3 (D) across Xist/Tsix locus in Dicerlox/lox (D3Cre) and Dicer deficient (S5, S6, E5) ES cell lines. Average data from three independent ChIP experiments is presented as percentage of input. [file 1756-8935-1-2-S3.pdf]

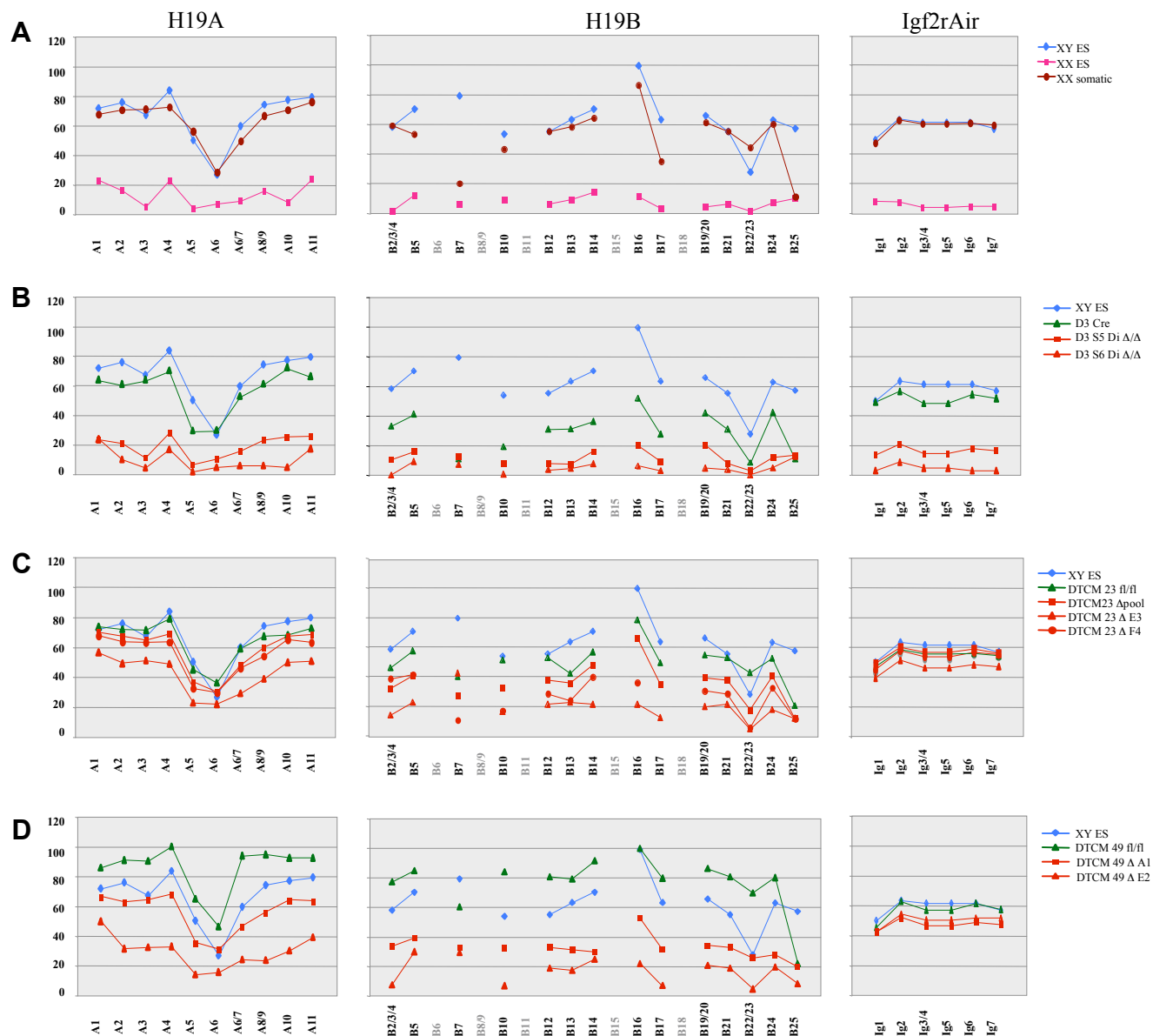

Supplement: Additional file 5 — SEQUENOM mass spectrometry analysis of DNA methylation of imprinted genes in Dicer deficient XY ES cell lines. Graphs show the methylation level of specific CpG sites of H19 DMR and Igf2rAir DMR in controls (A) (XY and XX ES cell lines and XX somatic cells) and three groups of Dicerlox/lox and deficient ES cell lines (B-D). Average data for at least three independent DNA samples is shown for each CpG site. The wt 129/1 XY ES cell line is included as a reference control on each graph. Dots are joined by lines when consecutive sites were analysed. Grey site numbers below the graphs indicate that the data points are not available due to low or high fragment mass or due to duplication or overlay of two or more fragments. The average data for two or three CpG sites (e.g. A6/7) is shown in cases when the sites reside close to each other and could not be resolved to separate fragments. [file 1756-8935-1-2-S5.pdf]
