# Supplementary material for: Dicer regulates Xist promoter methylation in ES cells indirectly through transcriptional control of Dnmt3a
Source: Epigenetics Chromatin. 2008 Oct 27;1:2. doi: 10.1186/1756-8935-1-2 (PMC2577046; doi:10.1186/1756-8935-1-2)
Supplement: Additional file 4 — Supplementary methods: Chromatin Immunoprecipitation. File includes Supplementary method for Chromatin Immunoprecipitation, control primers and PCR conditions used for ChIP analysis and supplementary references. [file 1756-8935-1-2-S4.doc]

**Additional file 4.** **Supplementary methods: Chromatin Immunoprecipitation**

Histone modifications were analysed by chromatin immunoprecipitation (ChIP) essentially as previously described (O'Sullivan and Martens 2005) with some modifications. Briefly, confluent ES cells were trypsinised and washed with ice-cold PBS. The cells were fixed in 1% formaldehyde solution in 1x ChIP fix buffer (0.5mM HEPES, pH 8.0; 1mM NaCl; 10μM EDTA; 5μM EGTA) in PBS for 10 min at room temperature with constant rotation. The crosslinking reaction was quenched by addition of 1/10 volume of 1.25M glycine. After three washes in cold PBS, the cells were lysed in ChIP lysis buffer (50mM Tris, pH 8.0; 10mM EDTA; 1% SDS), containing protease inhibitors (Complete mini, Roche Diagnostic). The lysates were sonicated using a Bioruptor sonicator until the fragment size was between 500-1000bp and stored at +4^0^C until immunoprecipitation. 5μg of antibody and 140μg of sonicated chromatin were used per immunoprecipitation. Antibodies for H3K4me2, H3K27me3 and H3K9me2 were purchased from Millipore, the H4K20me3 antibody was from Abcam and the anti-mouse IgG used as negative contol was from Sigma. Primers and PCR conditions are given in Table 2 and supplementary Table below.

Primers and PCR conditions for quantitative PCR

| Region/Gene | Primer, forward | Primer, reverse | PCR conditions |
| --- | --- | --- | --- |
| Oct4 promoter | Oct4_F1, ggctctccagaggatggctgag | Oct4_R1,  tcggatgccccatcgca | 95^0^C 3min; (95^0^C 20s; 60^0^C 20s)x40 |
| Oct4 exon | Oct_4F,  gaacctggctaagcttccaa | Oct_4R,  agttgctttccactcgtgct | 95^0^C 3min; (95^0^C 20s; 60^0^C 20s)x40 |
| Cdx2 promoter | Cdx2_F3, accaccttctgcctgagaatgtac | Cdx2_R3, cctccaatcacaggttcaaagact | 95^0^C 3min; (95^0^C 20s; 60^0^C 20s)x40 |
| Major repeats | Maj Sat_F1, gacgacttgaaaaatgacgaaatc | Maj Sat_R1, catattccaggtccttcagtgtgc | 95^0^C 3min; (95^0^C 20s; 60^0^C 20s)x40 |

Navarro, P., Pichard, S., Ciaudo, C., Avner, P., and Rougeulle, C. 2005. Tsix transcription across the Xist gene alters chromatin conformation without affecting Xist transcription: implications for X-chromosome inactivation. *Genes Dev* **19**(12): 1474-1484.
