# Supplementary material for: Dicer regulates Xist promoter methylation in ES cells indirectly through transcriptional control of Dnmt3a
Source: Epigenetics Chromatin. 2008 Oct 27;1:2. doi: 10.1186/1756-8935-1-2 (PMC2577046; doi:10.1186/1756-8935-1-2)
Supplement: Additional file 2 — SEQUENOM mass spectrometry analysis of Xist DNA methylation in Dicer deficient XY ES cell lines. Schematic representation of the Xist promoter region and the 5'end of exon 1 (CpG regions 1 and 2, see Fig. 2 legend for detailed description). The graphs show the percentage of methylation of specific Xist CpG sites in DTCM49 Dicerlox/lox and deficient ES cell lines (A). Average data for at least three independent DNA samples is shown for each CpG site. The wt 129/1 XY ES cell line is included as a reference control on each graph. The dots are joined by lines when consecutive sites were analysed. CpG sites numbered in grey below the graphs indicate that the data points are not available due to low or high fragment mass or due to duplication or overlay of two or more fragments. The average data for two or three CpG sites (e.g. A7/8/9) is shown in cases when the sites reside close to each other and could not be resolved as separate fragments. (B) Dynamic of Xist CpG island hypomethylation in DTCM49 floxed cell line exposed to tamoxifen for 50 hrs (blue) or 168 hrs (lilac). [file 1756-8935-1-2-S2.pdf]

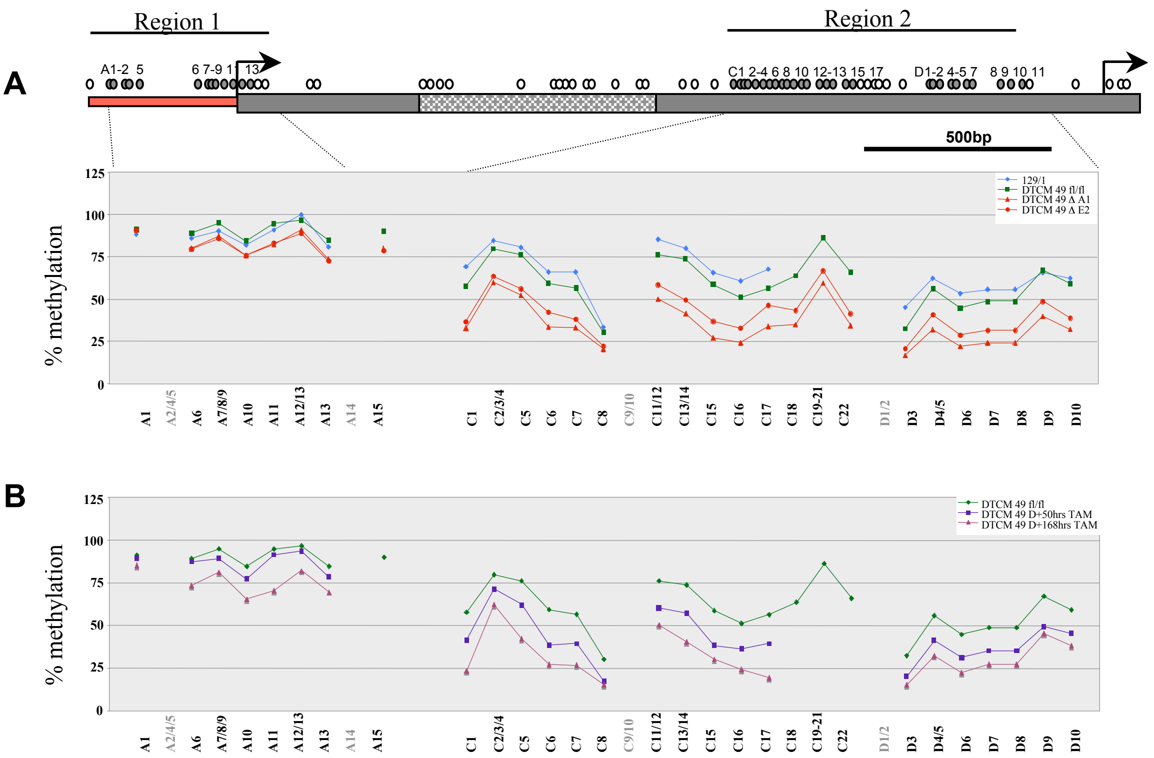
A
Region 1
Region 2
A1-2 5
6 7-9 11 13
C1 2-4 6 8 10 12-13 15 17
D1-2 4-5 7 8 9 10 11
500bp
% methylation
129/1
DTCM 49 fl/fl
DTCM 49 Δ A1
DTCM 49 Δ E2
A1
A2/4/5
A6
A7/8/9
A10
A11
A12/13
A13
A14
A15
C1
C2/3/4
C5
C6
C7
C8
C9/10
C11/12
C13/14
C15
C16
C17
C18
C19-21
C22
D1/2
D3
D4/5
D6
D7
D8
D9
D10
B
% methylation
DTCM 49 fl/fl
DTCM 49 D+50hrs TAM
DTCM 49 D+168hrs TAM
